# Multi-Modality Emotion Recognition Model with GAT-Based Multi-Head Inter-Modality Attention

**DOI:** 10.3390/s20174894

**Published:** 2020-08-29

**Authors:** Changzeng Fu, Chaoran Liu, Carlos Toshinori Ishi, Hiroshi Ishiguro

**Affiliations:** 1 Advanced Telecommunications Research Institute International, Kyoto 619-0288, Japan; chaoran.liu@atr.jp (C.L.); carlos@atr.jp (C.T.I.); ishiguro@sys.es.osaka-u.ac.jp (H.I.); 2 Graduate School of Engineering Science, Osaka University, Osaka 560-8531, Japan; 3 Interactive Robot Research Team, Robotics Project, RIKEN, Kyoto 619-0288, Japan

**Keywords:** emotion recognition, multi-modality, graph attention network

## Abstract

Emotion recognition has been gaining attention in recent years due to its applications on artificial agents. To achieve a good performance with this task, much research has been conducted on the multi-modality emotion recognition model for leveraging the different strengths of each modality. However, a research question remains: what exactly is the most appropriate way to fuse the information from different modalities? In this paper, we proposed audio sample augmentation and an emotion-oriented encoder-decoder to improve the performance of emotion recognition and discussed an inter-modality, decision-level fusion method based on a graph attention network (GAT). Compared to the baseline, our model improved the weighted average F1-scores from 64.18 to 68.31% and the weighted average accuracy from 65.25 to 69.88%.

## 1. Introduction

For decades research interest in emotion recognition has increased. In social interactions, emotion perception is critical in decision handling for humans and artificial agents. The performance of artificial agents would improve in human–agent interaction if they had good emotion recognition ability with appropriate conduct [1,2,3]. Centered on this topic, much research has examined facial expression recognition [4,5], speech emotion recognition [6,7], motion emotion recognition [8,9], and text emotion recognition [10].

Previous studies [11,12,13,14] on emotion recognition were mainly based on datasets (e.g., EMOVO [15], DaFEx [16], CASIA [17]) that invited actors to perform specific emotions. However, models trained on those datasets might not be suitable for implementation in daily practical interactions since the training data were sampled from intentional emotions that express the performances of actors where the emotions are stronger and more infectious than people’s daily behaviors. Therefore, researchers in this field have turned their research focus to natural human–human interaction [18,19,20] on the interactive emotional dyadic motion capture database (IEMOCAP) [20]. However, from the results reported by previous studies, the accuracy of emotion recognition based on a single modality remains inadequate. Naturally, emotion perception by humans is not decided by just one type of information; it is triggered by a multitude of factors or signals emitted from others. By investigating such factors, many researchers have proposed multi-modality approaches to improve the performance of emotion recognition [21,22,23,24,25,26].

Despite the achievements reached by multi-modality emotion recognition models, it remains unclear what exactly is the most appropriate way to fuse the features from different modalities, so that the model can perfectly leverage the variant strengths of the modalities to enhance the performance of emotion recognition.

In this paper, we report such strategies as audio samples augmentation and an emotion encoderdecoder that we employed to improve the performance of the uni-model for emotion recognition. We also propose a graph attention network (GAT) for constructing a decision-level fusion strategy. We evaluated our proposed method on IEMOCAP [20] and compared the evaluation results of two baselines, DialogueRNN [23] and DialogueGCN [27], which are the most recent studies that report state-of-the-art results. The following are the contributions of this study:

- We propose a strategy for augmenting audio samples based on the entropy weight method.
- We propose an emotion encoder-decoder to select decisive timesteps.
- We propose a multi-modality emotion recognition model that combines visual, audio, and text modalities.
- We propose a decision-level fusion strategy with a graph attention network.

The rest of this paper is organized as follows. Related works for emotion recognition are introduced in Section 2. Section 3 describes our proposed method. Section 4 and Section 5 demonstrate the setting of our experiment and provide our comparison results with the state-of-the-art models. Finally, we discuss our findings and conclude in Section 6 and Section 7.

## 2. Related Works

### 2.1. Emotion Recognition with Deep Learning Approach

#### 2.1.1. Facial Expression Recognition (FER)

Facial expression is the most direct way we humans express our mental states. The goal of facial expression recognition is to identify the discriminative and distinguishable features of a face. In this task, a lot of deep learning-based methods with high performances have been proposed. Researchers mainly utilized convolutional neural networks (CNNs) to build facial expression recognition models due to their good performances and extensive adoptions in diverse computer vision applications [28,29]. Many studies improved the performance of facial expression recognition with such pre-trained models, including VGG-face [30], ResNet [31]. It has been well known that the emotion in a certain moment is greatly influenced by the past moments [23], but the above studies all used 2D-CNN to recognize facial expression at the image/frame level. To deal with this defect of overlooking the time-series information and improve the performance of facial expression recognition, researchers began to employ 3D-CNN to process the time series of emotional data from video [5,23,32,33]. Various attention mechanisms have been integrated into 3D-CNN to capture the timesteps that mostly contribute to the determination of emotion [34,35]. Based on the benefits of such previous research, we employed a pre-trained 3D-CNN to capture the facial spatial-temporal features.

#### 2.1.2. Speech Emotion Recognition (SER)

People can generally perceive the emotions of others from speech. Such an ability maintains inter-personal relationships. Speech emotion recognition has become a very popular research topic for achieving artificial intelligence and improving human–agent interaction. In this field, researchers mainly focus on discriminative emotion features and recognition models. For feature extraction, some studies sought the appropriate speech duration [36], extracted suitable audio feature sets [37], and tested whether specific prosodic events offered great discriminative contributions to emotion recognition [38]. With the development of neural networks, some studies constructed novel models with recurrent neural networks (RNNs) and convolutional neural networks (CNNs) [39,40], both of which achieved excellent performance on speech emotion recognition tasks. These related studies on speech emotion recognition show that selecting features and decisive durations improve the performance of SER models. In this study, we follow such effective clues to propose an emotion encoder-decoder (EED).

#### 2.1.3. Text Emotion Recognition

Detecting emotions in a text is challenging since there is a lack of information on facial expressions and speech. Deep learning-based approaches are frequently employed in text emotion recognition tasks and successfully predicted emotions in TV transcripts [41], identified the emotions of tweets [42] and comments [43]. However, the methods proposed in the above references just predicted or detected the emotions based on every sentence. In real-world interactive applications, artificial intelligence agents need the ability to detect and predict users’ emotions in conversations/dialogues, where the influence of contextual information is critical. To utilize contextual information in dialogues for detecting emotions, Hazarika et al. [44] employed two distinct gate recurrent networks (GRUs) for different speakers and fed two GRUs with the utterance context from the dialogue. Majumder et al. [23] and Ghosal et al. [27] constructed a model that separately analyzed the global emotion states and party states from dialogue contexts and calculated the effects of the interlocutors and individual historical utterances to detect emotions. However, in practical implementations, since agents hard to express emotions affect users, an effective model is required that can focus on the mental state trends of only the users in the dialogue. In our study, we refer to the user’s utterance at the last moment to facilitate the emotion recognition of the current/incoming utterance.

### 2.2. Multi-Tasks

Since a multi-task strategy can introduce multi-loss learning advantages to a model, this excellent strategy has been widely implemented on emotion recognition tasks [24,45,46,47,48,49,50]. Kollias et al. [47] proposed a facial expression recognition model that simultaneously detected actions, expressions, and valence to achieve a good performance. For speech emotion recognition tasks, researchers always treated both categorical and dimensional emotion recognition as multi-task learning [24,48,49,50]. Some special configurations set gender recognition, emotion recognition, and type/genre recognition as multi-tasks to distinguish thresholds from distributions in training samples [49]. In our study, we set gender classification and emotion recognition as multi-tasks to differentiate the natural differences of acoustic features between males and females.

### 2.3. Multi-Modality

Naturally, the emotion perception of humans is not just determined by one type of information; it is triggered by a multitude of factors or signals emitted from others. Many studies have utilized multimodality (i.e., visual, audio, and text) to improve the performance of emotion recognition [19,21,22,23,51]. Zhou et al. [51] and Tripathi et al. [19] modeled the relationships among text, visual, and audio modalities by deep learning methods to improve performance. Majumder et al. [23] used the contextual multi-modality information in a dialogue to detect human social emotions. In addition to the modalities that can be extracted from video, Asghar et al. [21] combined the electroencephalography (EEG) modality to facilitate the model’s performance. In our study, we combined text, visual, and audio modalities to construct a multi-modality emotion recognition model. Furthermore, other than simply merging the hidden features from different modalities, we adopted a graph attention network (GAT) so that different modal features can interact and leveraged the strength of each modality.

### 2.4. Graph Neural Networks

In the real world, many types of information can be constructed as non-grid-like topologies, such as social and traffic networks. Graph neural networks (GNNs) have emerged to deal with such information. At the same time, studies discovered that GNN can also promote the performance of emotion recognition [27,52]. Zhang et al. [52] recognized emotions with EEG-channel signals by a graph convolutional broad network (GCB-Net), which explored the deeper-level information of structured data. Ghosal et al. [27] adopted a graph convolutional network (GCN) to deeply capture the inter-relationship utterances/emotions in a dialogue. In our study, we regarded the hidden features from different modalities as a node and employed a graph attention network (GAT) to emphasize the strengths of each modality.

## 3. Proposed Methods

### 3.1. Preprocessing

In this subsection, we explain how we processed the samples from the audio, text, and visual modalities.

We first set the maximal length of each utterance to 6.5 s (the mean duration plus the standard deviation). The longer utterances were cut at 6.5 s, and the shorter ones were padded with zeros. The sampling rate was set to 16,000 Hz. For each frame, we calculated a Discrete Fourier Transform (DFT) of length 8000 with a hop length of 400. The scale of the Mel spectrogram was set to 384. Moreover, to balance the number of training samples, we designed an augmentation strategy based on the entropy weight method. Denoting $A\in R^{m\times n}$ as an extracted spectrogram, *n* is the number of timesteps and *m* is the number of the Mel scale. For augmentation, we normalized each element in *A* by Equation (1), where $x_{ij}$ stands for the original element in the Mel spectrogram and $p_{ij}$ is the normalized element. Then we used Equations (2) and (3) to obtain the augmented samples. The alpha in Equation (3) is a constant, which we set to 2:

(1) $$\begin{array}{c} p_{ij}=x_{ij}/\sum_{1}^{n}x_{ij}\;\;\;\;\left(j=1,2,\ldots ,m\right),\\ where\;\;\;x_{ij}=x_{ij}/max\left(A\right) \end{array}$$

(2) $$\begin{array}{c} e_{j}=-k\cdot \sum_{1}^{n}p_{ij}\cdot log\left(p_{ij}\right)\;\;\;\;\left(j=1,2,\ldots ,m\right),\\ where\;\;\;k=1/log(n) \end{array}$$

(3) $$\begin{array}{cc} \; & w_{j}=1-e_{j}/\sum_{1}^{m}\left(1-e_{j}\right)\\ \; & A_{new}=alpha\cdot W\cdot A. \end{array}$$

General speaking, the above equations rescale the original features of each scale of the Mel spectrogram to emphasize the features with large information entropy values.

The features with low information entropy are assigned a certain value without ignoring.

**Facial Expressions**: We extracted the faces from videos frame by frame (in each utterance) and selected ten frames with an average interval based on the length of every utterance. We followed the method of Majumder et al. [23] who employed a 3D-CNN [5] to extract spatial-temporal features. 3D-CNN is a pre-trained, facial expression recognition model that consists of two individual channels of input streams: a spatial CNN network branch that processes static frame-level cropped facial images and a temporal CNN network branch that processes optical flow images. After extracting the spatial-temporal features by these two branches, a fusion network integrates them. In our case, the features we extracted are the output of a hidden layer in the fusion network.

**Texts**: To convert each utterance transcript into a vector and tokenize the sentences, we adopted the WordPiece tokenizer, which is utilized in BERT [53]. With WordPiece, we split a word into its stem and tense, for example, ‘seeing’ to ‘see’ and ‘##ing’. After building a dictionary of tokens, we referred to the index of tokens to compose vectors for representing each utterance.

### 3.2. Emotion Encoder-Decoder (EED)

Each utterance’s emotion is jointly determined by the entire time-series information, and such sequence information has some key decisive timesteps. To consider the relationship among each timestep in a sample and select the decisive ones, we adopted an emotion encoder-decoder (EED), inspired by Bahdanau et al. [54] from the natural language translation field.

For the encoding stage, assume that the encoder’s input is matrix $X=\left[x_{1},x_{2},\ldots ,x_{t}\right],\;x_{t}\in R^{n_{f}}$. Equations (4) and (5) illustrate the flow that we calculate as hidden state $h_{i}$. $\vec{W}$, ${\vec{W}}_{z}$, ${\vec{W}}_{r}$$\in R^{n_{h}\times n_{f}}$, $\vec{U}$, ${\vec{U}}_{z}$, ${\vec{U}}_{r}$$\in R^{n_{h}\times n_{h}}$ are weight matrices. $n_{h}$ and $n_{f}$ are the hidden units and features obtained from the previous layer. Note that $n_{f}$ varies from each modality (i.e., audio and visual, see Table 1). Notations with forward arrows indicate forward processing time-series sequences, and backward arrows denote backward processing time-series sequences:

(4) $$\begin{array}{c} h_{i}=\left[{\vec{h}}_{i},{\overset{\leftarrow }{h}}_{i}\right], \end{array}$$

where the bias are omitted:

(5) $$\begin{array}{cc} \; & {\vec{h}}_{i}=\left(1-{\vec{z}}_{i}\right)\cdot {\vec{h}}_{i-1}+{\vec{z}}_{i}\cdot {\underline{\vec{h}}}_{i}\\ \; & {\underline{\vec{h}}}_{i}=tanh\left(\vec{W}x_{i}+\vec{U}\left[{\vec{r}}_{i}\cdot {\vec{h}}_{i-1}\right]\right)\\ \; & {\vec{z}}_{i}=\sigma \left({\vec{W}}_{z}x_{i}+{\vec{U}}_{z}{\vec{h}}_{i-1}\right)\\ \; & {\vec{r}}_{i}=\sigma \left({\vec{W}}_{r}x_{i}+{\vec{U}}_{r}{\vec{h}}_{i-1}\right) \end{array}$$

${\overset{\leftarrow }{h}}_{i}$ similarly.

For the decoding stage, $Eh\in R^{n_{\mathrm{ef}}}$ (Equation (6)) denotes the emotion hidden layer, and $n_{ef}$ is the length of the hidden emotion feature. We flattened the hidden states obtained from the encoding stage and employed a dense layer as a bottleneck to compute the hidden emotion vector.

### 3.3. Model

**Figure 1:**
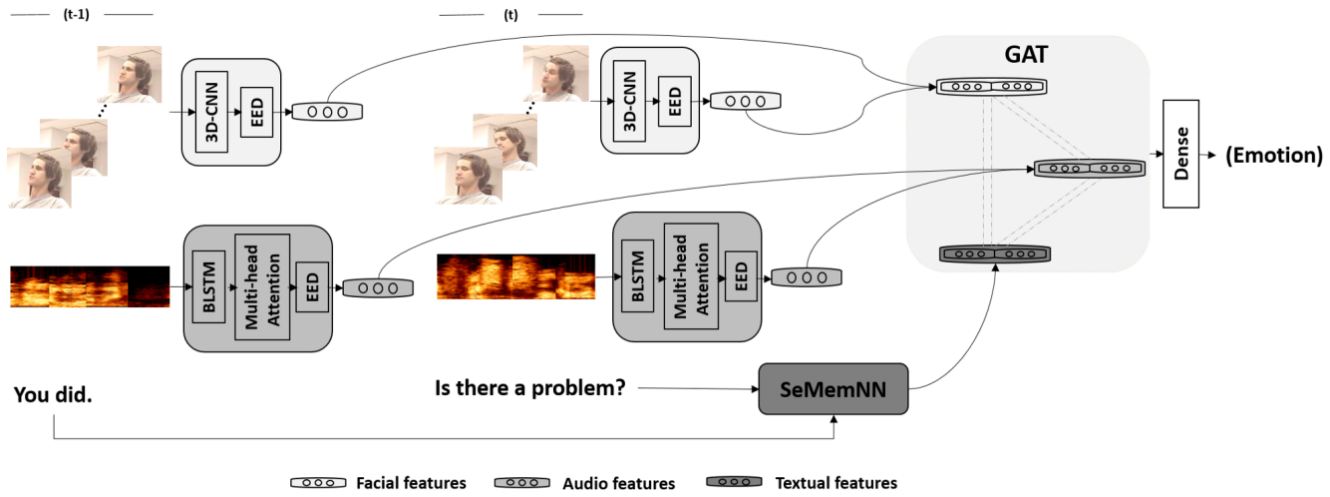
Architecture of proposed multi-model for emotion recognition: For the visual model, we used a 3D-convolutional neural network (CNN) to capture the affective time-spatial features and analyzed the emotions with an emotion encoder-decoder (EED). Audio is composed of a bidirectional long-short term memory layer (BLSTM), a multi-head attention layer, and an EED. Audio model treats the Mel spectrogram as input to predict the emotion. Text model employed a SeMemNN to analyze emotions from semantics. Note that the multi-model predicts emotions at time *t* by considering multi-modality data at times t−1 and *t*.

In this study, we implemented different neural networks according to each modality (Figure 1). Majumder et al. [23] investigated the effects of contextual information and clarified that the closer an utterance is to the target utterance, the greater its contribution. Therefore, we regarded the identical speaker’s data at timesteps *t* and t−1 as the inputs of each uni-model. The uni-models were trained separately. We then extracted the last hidden layer (the hidden emotion vector) of each model and fine-tuned the combined model after each uni-model was well trained.

**Audio**: We first separately fed the extracted Mel spectrogram at timesteps t−1 and *t* to a bidirectional long-short term memory layer (BLSTM) to process the time-series information and then employed a multi-head attention layer to emphasize in parallel the relationship among the timesteps. After that, the emphasized hidden states are passed to the EED, which selects the information that contributes most to determine the emotion. A multi-task training strategy was adopted for the audio model to improve the performance. In addition to classifying the emotion label, our model also recognized the speaker’s gender at the same time in a way to enhance it by considering the impact of the pitch variations between women and men.

**Facial Expressions**: After extracting the speakers’ faces from the videos, we separately fed the data to the 3D-CNN [5] to obtain a hidden vector that contains the spatial-temporal features. Apart from the benefits of extracting relevant features from each image frame, this 3D-CNN also extracts the spatiotemporal features by separately considering the facial feature in each frame (spatial branch) and the optical flow across the frames (temporal branch). In the procedure of analyzing the facial expressions, a VGG model is adopted to individually fine-tune the spatial branch. The temporal branch straightforwardly processes the entire video message where the input is a video of frames, height, and weight dimensions; 3 represents the RGB channels. To deeply fuse the learned spatiotemporal CNN features, we employed a fully connected neural network to jointly learn the discriminative features. Then the EED processed the features and selected the decisive information to predict the emotion.

**Text**: For the text emotion classification, we employed SeMemNN [55] and trained it from scratch. Fu et al. [55] confirmed that SeMemNN has a good ability to learn semantics and fast training speed on a small sample dataset. In SeMemNN, two inputs work together to construct the addressing and semantic matrixes, which yield an address vector to read some corresponding information from the semantic matrix. Based on the SeMemNN structure, the text at timesteps t−1 and *t* yields the addressing matrix, and the semantic matrix was individually generated by the text at timestep *t*.

**Multi-model**: Each uni-model for the audio, visual, and text modalities was trained separately under the emotion recognition task in an end-to-end manner. After the uni-models were well trained, we extracted the hidden features from each modality and employed a multi-head GAT to compute the inter-modality relationship among the three modalities. Equation (6) generally demonstrates how this strategy works, where *K* is the number of attention heads, W is the weight for the learnable linear transformation, $a\in R^{2n_{f}}$ is a weight vector, and *N* equals 3 (i.e., the audio, text, and visual modality nodes). GAT’s multi-head design establishes multiple channels to capture the relationship among the audio, visual, and text modalities for different emotions. Since this operation among the modalities and each modality is regarded as a node, we named it the multi-head inter-modality graph attention.

(6) $$\begin{array}{cc} \; & Eh_{i}=\sigma \left(\frac{1}{K}\sum^{K}\sum_{j\in N}\alpha_{ij}^{k}W^{k}Eh_{j}\right)\\ \; & \alpha_{ij}=\frac{exp\left(LeakyReLU\left(a^{T}\right)\right)\left[WEh_{i}∥WEh_{j}\right]}{\sum_{j\in N}exp\left(LeakyReLU\left(a^{T}\right)\right)\left[WEh_{i}∥WEh_{j}\right]}. \end{array}$$

## 4. Experiment

In this section, we demonstrate our experiment’s setting, the dataset with which we evaluated our proposed method, and the baselines with which we compared the performances.

### 4.1. Dataset

We evaluated our proposed method on an emotion detection dataset: the interactive emotional dyadic motion capture database (IEMOCAP) [20]. This dataset contains videos of two-way conversations with ten speakers. Each video contains a single dyadic dialogue that is segmented into utterances and annotated with emotion labels. We strictly follow Majumder et al. [23] and Ghosal et al. [27] and split the datasets into training and testing sets with a rough 80/20 ratio: 5810 training samples and 1623 testing samples.

### 4.2. Setting

Our proposed model was implemented with Keras and set adam as the optimizer where the learning rate was set to 1e−4. We set the number of cells for BLSTM to 128 and implemented multi-head attention with eight heads. For the audio model, the weights for the loss function in each task equaled 1. The other hyper-parameters are shown in Table 1. The numbers of trainable parameters of the audio, visual, and text models are around 13.6, 13.3, and 24.8 million, respectively. The number of trainable parameters for the final multi-model is 51.8 million.

### 4.3. Baseline

We compared the benefits produced by our proposed methods by setting different configurations and made the following self-comparing and external comparisons with the benchmarks:

- Without audio sample augmentation: We trained the audio model with and without augmentation samples and compared and analyzed the performances.
- Replacing EED with LSTM: The internal mechanism of our proposed EED resembles LSTM, but we added more trainable matrices *U* to analyze the time-series information and learn the linear transformation of each timestep. We therefore trained a model for comparison, in which the EED was replaced with a bi-directional LSTM.
- Comparing SeMemNN to BERT: Although BERT’s superior performance is recognized in the field of text classification, few studies have applied BERT to dialogue text emotion recognition. In our study, we adopted SeMemNN, which performs well on small-sample training, and compared its performance to a pre-trained BERT [53].

Regarding multi-model comparison, we chose two state-of-the-art studies as baselines:

- DialogueRNN [23]: This recurrent network adopts two GRUs to track the individual speaker states by considering the global context during the conversation and uses another GRU to track the flow of the emotional states.
- DialogueGCN [27]: This method employs a graph convolutional network (GCN) to leverage the self- and inter-speaker dependency of the interlocutors with contextual information.

## 5. Results

In this section, we first separately present the results of each uni-model and then those of the proposed multi-model. When describing each result, we present the results of the corresponding comparison experiment as well.

### 5.1. Speech Emotion Recognition

**Figure 2:**
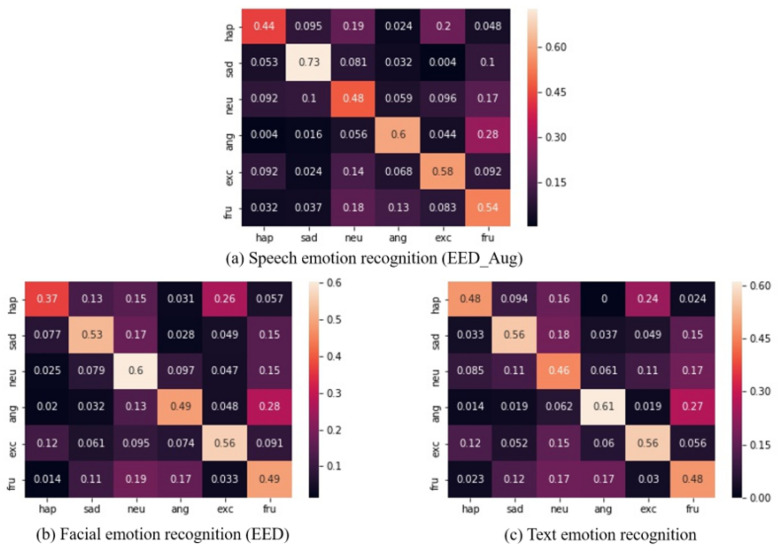
Confusion matrices for proposed uni-models: Columns are ground-truth, and rows are predictions.

Table 2 shows the results of the audio models with different configurations. AM denotes the audio model (the speech emotion recognition model). The suffix EED stands for an audio model with EED, and LSTM indicates an audio model with LSTM. Aug means that the training data were augmented, and Ori indicates that the model was trained on the original data without augmentation. The bold numbers in the table are the highest accuracies. The audio model with EED and the one trained on augmentation data achieved better performances than the other configurations. Regarding the comparison between the audio model with EED and the one with LSTM, when trained on the data with augmentation, the EED has advantages in *happy*, *sad*, *excited*, and *frustrated* around 8.15%, 7%, 1.31%, and 6.79%. It also improves the weighted average accuracy from 54.04% to 56.30% and the F1-scores from 53.34% to 55.73%. When trained on the original data, the EED improves the accuracy of *neutral*, *angry*, *excited*, and *frustrated* with around 9.86%, 0.57%, 13.92%, and 5.47% values, the weighted average accuracy improved from 47.87% to 52.11%, and the F1-score increased from 46.91% to 51.21%. When the augmentation method employed the EED, it has advantages over *happy*, *sad*, *neutral*, *angry*, and *frustrated* at around 3.67%, 15.12%, 0.79%, 0.83%, and 9.67%; it improved the weighted average accuracy from 52.11% to 56.30% and the F1-scores from 51.21% to 55.73%. When employing the LSTM, the augmentation method improved the accuracy of *sad, neutral, angry, excited*, and *frustrated* by around 0.46%, 11.8%, 8.42%, 4.41%, and 8.17%, the weighted average accuracy improved from 47.87% to 54.04%, and the F1-scores increased from 46.91% to 53.34%. Generally, our proposed augmentation method significantly improved the accuracy of *sad and frustrated*, regardless of whether the model used EED or LSTM. The audio model with EED has advantages over *happy*, *sad*, and *frustrated* regardless whether the training data are augmented. Finally, the audio model with EED and the proposed augmentation strategy achieved the highest accuracy for *happy*, *sad*, and *frustrated* and the average accuracy as well as the F1-scores among all the configurations. Figure 2a is the confusion matrix for audio model AM${}_{EED\_Aug}$.

### 5.2. Facial Expression Recognition

Table 3 presents the results of the visual models. VM is the notation for the visual model (facial expression recognition model). The suffix EED stands for a visual model with EED, and LSTM stands for a visual model that replaces EED with LSTM. Our results show that the visual model with EED achieved a better performance. EED increased the accuracy of *sad*, *neutral*, *angry*, and *excited* by about 5.22%, 4.01%, 6.17%, and 9.69% and also has advantages over the weighted average accuracy and the F1-scores of around 1.48% and 3.34%. Despite the improvements, $VM_{EED}$’s performance for happy and frustrated still greatly lags behind the accuracy achieved by $VM_{LSTM}$. Regardless whether we use LSTM or EED, a visual model always has an advantage identifying neutral emotions. Figure 2b is the confusion matrix for visual model with EED.

### 5.3. Text Emotion Recognition

Table 4 shows the results of the text models. $TM_{SeMemNN}$ denotes a text model that consists of SeMemNN, which was trained from scratch, and $TM_{BERT}$ stands for a text model that consists of an English pre-trained BERT. SeMemNN significantly outperformed BERT and increased the weighted average accuracy from 39.62% to 52.99% and the F1-scores from 36.13% to 51.51%. Moreover, based on the results of each emotion label, SeMemNN has an advantage in the text emotion recognition on the IEMOCAP dataset compared to BERT because it increased the accuracy of each emotion category by at least 10%. SeMemNN also improved the accuracy of the angry label by around 30 points, raising it to the best rank among the six categories. BERT is not good at recognizing angry emotions. Figure 2c is the confusion matrix for text model with SeMemNN.

### 5.4. Multi-Modality

Naturally, how humans perceive emotions is not only decided by one type of information. Such perception is triggered by a multitude of factors or signals from others. Furthermore, from the confusion matrices of the audio, visual, and text models, their respective strengths in recognizing emotions are different. More specifically, the proposed audio model deftly detects sad emotions, the visual model has an excellent ability to recognize neutral emotions, and the text model performs better with angry expressions than the other labels. Establishing a multi-model can combine the strengths of various modalities and improve the performance of emotional recognition.

The multi-model was integrated with the best uni-models (i.e., $VM_{EED}$, $AM_{EED\_Aug}$ and $TM_{SeMemNN}$). Table 5 shows the comparison results, where the bold font denotes the best performance. The multi-model without GAT achieved a weighted average accuracy of 67.26% and a weighted average F1-score of 66.74%, which are increases of about 2% from the 65.25% and 64.18% results achieved by DialogueGCN. Such a result is around 5.5% better than DialogueRNN. Moreover, after adopting a GAT to implement the inter-modality attention, our model achieved a weighted average accuracy of 69.88% and a weighted average F1-score of 68.34%, which is around 2.5% better than the multi-model without GAT. Regarding the results for each emotion label, our model improved the performances on happy, neutral, angry, as well as frustrated. Among these improvements, a huge gap appears for the happy label: 47% higher than DialogueGCN. Furthermore, compared to the multi-model without GAT, the one with GAT has advantages of at least 5% for happy, angry, and frustrated.

**Figure 3:**
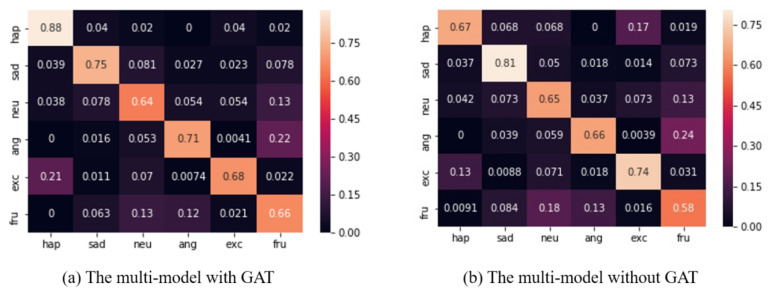
Confusion matrices for proposed multi-model with and without graph attention network (GAT): Columns are ground-truth, and rows are predictions.

Figure 3 presents a confusion matrix of the proposed multi-model. The proposed model misclassifies several samples of neutral, angry, and frustrated with and without GAT as the model. We found a very similar misclassification of happy and excited. However, this is a mutual misclassification of the model without GAT; the model with GAT just misclassified the excited samples as happy.

Additionally, we also added some cross-validation to completely evaluate our model under the same training-test ratio. The training-testing splitting still followed the leave-one-session-out strategy, the evaluation hereby became 5-folder cross-validation. The final weighted accuracy and F1-scores were 68.78±1.35 and 67.84±0.83, respectively. The result implies that our model is robust and has good generalizability.

## 6. Discussions

In previous sections, we reported the results of both uni-model and multi-model emotion recognition. In this section, we scrutinize our results.

**Audio**: The proposed augmentation method rescales the features of the spectrogram based on the entropy weight method, allowing the audio model to more easily capture the key information to represent emotions. Moreover, the EED we proposed has been confirmed to have better ability to discover decisive factors than LSTM. Despite the advantages introduced by our proposed methods, two remaining shortcomings must be addressed. The first one is that the audio model performs more poorly on *excited* after being trained on augmented data. This result may be caused when our augmentation method erases some information about excited emotions. When the excited emotions are expressed, the MFCCs, energy, and F0 values may temporarily increase, erasing or weakening the information of the cross-entropy weight on the timestep that the spectrogram should be contained. Another limitation is that the audio model with LSTM performs better on *angry* than the audio model with EED. We will analyze potential explanations in future work.

**Facial Expressions**: From Figure 2b, the misclassification of the visual model on neutral is mainly concentrated in frustrated, although the misclassification results of the other two modalities are decentralized, indicating that the visual model’s contribution to the multi-model mainly lies in detecting neutral emotions.

**Text**: Figure 2c shows that the recognition results of such intense emotions are more aggregated as happy, angry, excited, and sad rather than being dispersed among too many confusing labels. Their misclassifications are concentrated in one or two confusing labels, and neutral and frustrated have a large portion of misclassified samples in multiple categories. Such serious dispersion basically does not exist in the other modalities. Perhaps this phenomenon is caused by the text of the intense emotions, where some words are directly associated to emotions, which greatly help the model determine emotions. However, there might be a lack of words that directly point to neutral emotions. Concerning the recognition model, BERT did not perform well on IEMOCAP’s text emotion recognition. Similar results were also reported by Ying et al. [56] and Kant et al. [57]. They claimed that transformer-based models like BERT perform well on some datasets but not on models that are unstable in the dialogue text emotion recognition. This situation probably results from the uneven lengths of the dialogue texts, and the short ones may be particularly brief. Another reason might be the unbalanced samples for fine-tuning, causing the model to overfit to the classes that are more represented in the dataset. Future research will explore ways to improve the performance of our transformer-based models on text emotion recognition.

**Multi-model**: The results between the model with/without GAT indicate that GAT helps fuse the emotional features from different modalities. Numerous past studies on emotion recognition tasks have simply concatenated the hidden vectors from different modalities at the decision level, which combines the features from different sources. Under such a strategy, the features equally contribute to determine the labels. However, features from different modalities should interact with and compensate for each other. The fusion strategy proposed in our study obeys the natural mechanism, which regards the features of different modalities as nodes and lets GAT capture the relationships among them to efficiently leverage their strength from modalities. This may explain why GAT improved the weak performance of some labels, e.g., happy and frustrated. Additionally, GAT seems to balance the decision for similar samples. For instance, GAT generally avoids assigning happy and excited to a certain label, but makes a balanced state for the decision making. This ability is also shown in the global results. Discarding the best performing accuracy, the average result of the model with GAT is better than without GAT (STD=5.96 for the model without GAT and STD=4.27 for the model with it). Thus, for most labels, no big gap among them is shown. Despite the good results achieved by the proposed method with GAT, it also has limitations. GAT is expected to exploit different modalities to compensate for the weakness of some specific labels. However, we observed that the model decreases the accuracy of some labels while increasing similar ones. The same phenomenon also appears between DialogueRNN and DialogueGCN (e.g., *happy* and *excited*). This question deserves further exploration and improvement.

As for the time consumption for our model. Although this model looks relatively large, its time consumption is acceptable. In our experiment, we used a GeForce RTX 2070 GPU for training. The time cost for each step was 264 ms, and each step contained a batch size of 16 samples. For practical applications of interaction, we mainly considered the conversational interaction scenarios between humans and robots. In this case, our system obtained the information of audio, text, and visual modalities. Therefore, our model can produce a relatively better performance than other existing technologies. Its time consumption to predict emotions is acceptable. Of course, in real applications, since the user’s behavior is not controlled, sometimes visual information will be lost. The speech to text accuracy will also be affected by environmental noise. In such cases, our proposed model might collapse, which is a limitation of this study and needs other technologies that offer supplementary support.

## 7. Conclusions

We proposed an augmentation method for audio samples, an emotion encoder-decoder, and a multi-modality emotion recognition model with a GAT-based decision-level fusion. Our statistical results proved that our proposed augmentation method and emotion encoder-decoder are useful. These results suggest that the emotion recognition model would benefit from a preprocessing method that re-scale the feature or a model that can emphasize features that contain more information. Moreover, the pre-trained BERT did not outperform SeMemNN on text emotion recognition. We also discussed GAT’s usefulness for inter-modality attentive future fusion based on the proposed multi-model’s results and showed that the proposed multi-model outperformed the baselines. The advantages of GAT-based multi-head inter-modality attention imply that much information can be interacted between/among modalities to improve the performance of a neural network model.

## Figures and Tables

**Table 1:** Notation details.

Notation	Description	Value
*m*	Mel scale	384
*n*	length of Mel spectrogram	256
$n_{f_{audio}}$	number of audio features	256
$n_{f_{facial}}$	number of facial features	64
$n_{ef}$	number of hidden emotion features	64
$n_{h}$	number of EED cells	128
*K*	attention heads for GAT	8

**Table 2:** Comparison results of audio model. The bold numbers in the table are the highest accuracies.

Methods	Happy	Sad	Neutral	Angry	Excited	Frustrated	Acc.(w)	F1(w)
AM${}_{EED\_Aug}$	**44.05**	**72.87**	47.95	60.00	58.25	**53.74**	**56.30**	**55.73**
AM${}_{EED\_Ori}$	40.38	57.75	47.16	59.17	**66.45**	44.07	52.11	51.21
AM${}_{LSTM\_Aug}$	35.90	65.87	**49.10**	**67.02**	56.94	46.77	54.04	53.34
AM${}_{LSTM\_Ori}$	42.11	65.41	37.30	58.60	52.53	38.60	47.87	46.91

**Table 3:** Comparison results of visual model. The bold numbers in the table are the highest accuracies.

Methods	Happy	Sad	Neutral	Angry	Excited	Frustrated	Acc.(w)	F1(w)
VM${}_{EED}$	37.11	**52.63**	**60.43**	**48.81**	**56.28**	49.04	**52.29**	**51.52**
VM${}_{LSTM}$	**50.00**	47.38	56.42	42.64	46.59	**55.66**	50.81	48.18

**Table 4:** Comparison results of text models. The bold numbers in the table are the highest accuracies.

Methods	Happy	Sad	Neutral	Angry	Excited	Frustrated	Acc.(w)	F1(w)
TM${}_{SeMemNN}$	**48.24**	**55.51**	**46.10**	**61.24**	**56.22**	**48.13**	**52.99**	**51.51**
TM${}_{BERT}$	34.43	44.29	30.76	31.90	55.13	38.79	39.62	36.13

**Table 5:** Comparison results of multi-models. The bold numbers in the table are the highest accuracies.

Methods	Happy	Sad	Neutral	Angry	Excited	Frustrated	Acc.(w)	F1(w)
DialogueRNN	25.69	75.10	58.59	64.71	**80.27**	61.15	63.40	62.75
DialogueGCN	40.62	**89.14**	61.92	67.53	65.46	64.18	65.25	64.18
– *our model*
MulM${}_{Dense}$	66.99	80.73	**64.66**	65.88	74.34	57.63	67.26	66.74
MulM${}_{GAT}$	**88.00**	75.19	64.07	**70.78**	68.27	**66.40**	**69.88**	**68.31**

## References

[1] Scheutz M., Schermerhorn P., Kramer J., Anderson D. (2007). First steps toward natural human-like HRI. Auton. Robot..

[2] Gonsior B., Sosnowski S., Mayer C., Blume J., Radig B., Wollherr D., KÃhnlenz K. Improving aspects of empathy and subjective performance for HRI through mirroring facial expressions. Proceedings of the 2011 RO-MAN.

[3] Fu C., Yoshikawa Y., Iio T., Ishiguro H. (2020). Sharing Experiences to Help a Robot Present Its Mind and Sociability. Int. J. Soc. Robot..

[4] Byeon Y.H., Kwak K.C. (2014). Facial expression recognition using 3d convolutional neural network. Int. J. Adv. Comput. Sci. Appl..

[5] Zhang S., Pan X., Cui Y., Zhao X., Liu L. (2019). Learning affective video features for facial expression recognition via hybrid deep learning. IEEE Access.

[6] Lotfian R., Busso C. (2019). Curriculum learning for speech emotion recognition from crowdsourced labels. IEEE/ACM Trans. Audio Speech Lang. Process..

[7] Fu C., Dissanayake T., Hosoda K., Maekawa T., Ishiguro H. Similarity of Speech Emotion in Different Languages Revealed by a Neural Network with Attention. Proceedings of the 2020 IEEE 14th International Conference on Semantic Computing (ICSC).

[8] Ahmed F., Gavrilova M.L. (2019). Two-layer feature selection algorithm for recognizing human emotions from 3d motion analysis. Proceedings of the Computer Graphics International Conference.

[9] Ajili I., Mallem M., Didier J.Y. (2019). Human motions and emotions recognition inspired by LMA qualities. Vis. Comput..

[10] Hazarika D., Poria S., Zimmermann R., Mihalcea R. (2019). Emotion Recognition in Conversations with Transfer Learning from Generative Conversation Modeling. arXiv.

[11] Chetty G., Wagner M., Goecke R. A multilevel fusion approach for audiovisual emotion recognition. Proceedings of the AVSP.

[12] Ratliff M.S., Patterson E. Emotion recognition using facial expressions with active appearance models. Proceedings of the HRI.

[13] Wang K., An N., Li B.N., Zhang Y., Li L. (2015). Speech emotion recognition using Fourier parameters. IEEE Trans. Affect. Comput..

[14] Chao L., Tao J., Yang M., Li Y. Improving generation performance of speech emotion recognition by denoising autoencoders. Proceedings of the 9th International Symposium on Chinese Spoken Language Processing.

[15] Costantini G., Iaderola I., Paoloni A., Todisco M. (2014). Emovo corpus: An italian emotional speech database. Proceedings of the International Conference on Language Resources and Evaluation (LREC 2014).

[16] Battocchi A., Pianesi F., Goren-Bar D. (2005). Dafex: Database of facial expressions. Proceedings of the International Conference on Intelligent Technologies for Interactive Entertainment.

[17] Pan S., Tao J., Li Y. (2011). The CASIA audio emotion recognition method for audio/visual emotion challenge 2011. Proceedings of the International Conference on Affective Computing and Intelligent Interaction.

[18] Satt A., Rozenberg S., Hoory R. Efficient Emotion Recognition from Speech Using Deep Learning on Spectrograms. Proceedings of the Interspeech.

[19] Tripathi S., Tripathi S., Beigi H. (2018). Multi-Modal Emotion Recognition on Iemocap with Neural Networks. arXiv.

[20] Busso C., Bulut M., Lee C.C., Kazemzadeh A., Mower E., Kim S., Chang J.N., Lee S., Narayanan S.S. (2008). IEMOCAP: Interactive emotional dyadic motion capture database. Lang. Resour. Eval..

[21] Asghar M.A., Khan M.J., Amin Y., Rizwan M., Rahman M., Badnava S., Mirjavadi S.S. (2019). EEG-Based Multi-Modal Emotion Recognition using Bag of Deep Features: An Optimal Feature Selection Approach. Sensors.

[22] Tsiourti C., Weiss A., Wac K., Vincze M. (2019). Multimodal integration of emotional signals from voice, body, and context: Effects of (in) congruence on emotion recognition and attitudes towards robots. Int. J. Soc. Robot..

[23] Majumder N., Poria S., Hazarika D., Mihalcea R., Gelbukh A., Cambria E. Dialoguernn: An attentive rnn for emotion detection in conversations. Proceedings of the AAAI Conference on Artificial Intelligence.

[24] Le D., Aldeneh Z., Provost E.M. Discretized Continuous Speech Emotion Recognition with Multi-Task Deep Recurrent Neural Network. Proceedings of the Interspeech.

[25] Sahu G. (2019). Multimodal Speech Emotion Recognition and Ambiguity Resolution. arXiv.

[26] Li J.L., Lee C.C. Attentive to Individual: A Multimodal Emotion Recognition Network with Personalized Attention Profile. Proceedings of the Interspeech.

[27] Ghosal D., Majumder N., Poria S., Chhaya N., Gelbukh A. (2019). Dialoguegcn: A graph convolutional neural network for emotion recognition in conversation. arXiv.

[28] Fasel B. Robust face analysis using convolutional neural networks. Proceedings of the Object Recognition Supported by User Interaction for Service Robots.

[29] Fasel B. Head-pose invariant facial expression recognition using convolutional neural networks. Proceedings of the Fourth IEEE International Conference on Multimodal Interfaces.

[30] Qawaqneh Z., Mallouh A.A., Barkana B.D. (2017). Deep convolutional neural network for age estimation based on VGG-face model. arXiv.

[31] He K., Zhang X., Ren S., Sun J. Deep residual learning for image recognition. Proceedings of the IEEE Conference on Computer Vision and Pattern Recognition.

[32] Tran D., Bourdev L., Fergus R., Torresani L., Paluri M. Learning spatiotemporal features with 3d convolutional networks. Proceedings of the IEEE International Conference on Computer Vision.

[33] Prasanna Teja Reddy S., Teja Karri S., Ram Dubey S., Mukherjee S. (2019). Spontaneous Facial Micro-Expression Recognition using 3D Spatiotemporal Convolutional Neural Networks. arXiv.

[34] Li H., Liu Q., Wei X., Chai Z., Chen W. (2019). Facial Expression Recognition: Disentangling Expression Based on Self-attention Conditional Generative Adversarial Nets. Proceedings of the Chinese Conference on Pattern Recognition and Computer Vision (PRCV).

[35] Du H., Zheng H., Yu M. Facial Expression Recognition Based on Region-Wise Attention and Geometry Difference. Proceedings of the Chinese Conference on Pattern Recognition and Computer Vision (PRCV).

[36] Tzinis E., Potamianos A. Segment-based speech emotion recognition using recurrent neural networks. Proceedings of the 2017 Seventh International Conference on Affective Computing and Intelligent Interaction (ACII).

[37] Rao K.S., Koolagudi S.G., Vempada R.R. (2013). Emotion recognition from speech using global and local prosodic features. Int. J. Speech Technol..

[38] Cao H., Benus S., Gur R.C., Verma R., Nenkova A. Prosodic cues for emotion: Analysis with discrete characterization of intonation. Proceedings of the 7th International Conference on Speech Prosody.

[39] An N., Verma P. (2015). Convoluted Feelings Convolutional and Recurrent Nets for Detecting Emotion From Audio Data.

[40] Trigeorgis G., Ringeval F., Brueckner R., Marchi E., Nicolaou M.A., Schuller B., Zafeiriou S. Adieu features? End-to-end speech emotion recognition using a deep convolutional recurrent network. Proceedings of the 2016 IEEE International Conference on Acoustics, Speech and Signal Processing (ICASSP).

[41] Zahiri S.M., Choi J.D. Emotion detection on tv show transcripts with sequence-based convolutional neural networks. Proceedings of the Workshops at the Thirty-Second AAAI Conference on Artificial Intelligence.

[42] Köper M., Kim E., Klinger R. IMS at EmoInt-2017: Emotion intensity prediction with affective norms, automatically extended resources and deep learning. Proceedings of the 8th Workshop on Computational Approaches to Subjectivity, Sentiment and Social Media Analysis.

[43] Li P., Li J., Sun F., Wang P. (2017). Short Text Emotion Analysis Based on Recurrent Neural Network. ICIE ’17: Proceedings of the 6th International Conference on Information Engineering.

[44] Hazarika D., Poria S., Zadeh A., Cambria E., Morency L.P., Zimmermann R. Conversational memory network for emotion recognition in dyadic dialogue videos. Proceedings of the 2018 Conference of the North American Chapter of the Association for Computational Linguistics: Human Language Technologies.

[45] Zhang Z., Wu B., Schuller B. Attention-augmented end-to-end multi-task learning for emotion prediction from speech. Proceedings of the ICASSP 2019—2019 IEEE International Conference on Acoustics, Speech and Signal Processing (ICASSP).

[46] Zhao H., Han Z., Wang R. Speech Emotion Recognition Based on Multi-Task Learning. Proceedings of the 2019 IEEE 5th Intl Conference on Big Data Security on Cloud (BigDataSecurity), IEEE Intl Conference on High Performance and Smart Computing, (HPSC) and IEEE Intl Conference on Intelligent Data and Security (IDS).

[47] Kollias D., Zafeiriou S. (2019). Expression, Affect, Action Unit Recognition: Aff-Wild2, Multi-Task Learning and ArcFace. arXiv.

[48] Xia R., Liu Y. Leveraging valence and activation information via multi-task learning for categorical emotion recognition. Proceedings of the 2015 IEEE International Conference on Acoustics, Speech and Signal Processing (ICASSP).

[49] Zhang B., Provost E.M., Essl G. Cross-corpus acoustic emotion recognition from singing and speaking: A multi-task learning approach. Proceedings of the 2016 IEEE International Conference on Acoustics, Speech and Signal Processing (ICASSP).

[50] Xia R., Liu Y. (2015). A multi-task learning framework for emotion recognition using 2D continuous space. IEEE Trans. Affect. Comput..

[51] Zhou J., Chen X., Yang D. (2019). Multimodel Music Emotion Recognition Using Unsupervised Deep Neural Networks. Proceedings of the 6th Conference on Sound and Music Technology (CSMT).

[52] Zhang T., Wang X., Xu X., Chen C.P. (2019). GCB-Net: Graph convolutional broad network and its application in emotion recognition. IEEE Trans. Affect. Comput..

[53] Devlin J., Chang M.W., Lee K., Toutanova K. (2018). Bert: Pre-training of deep bidirectional transformers for language understanding. arXiv.

[54] Bahdanau D., Cho K., Bengio Y. (2014). Neural machine translation by jointly learning to align and translate. arXiv.

[55] Fu C., Liu C., Ishi C., Yoshikawa Y., Ishiguro H. SeMemNN: A Semantic Matrix-Based Memory Neural Network for Text Classification. Proceedings of the 2020 IEEE 14th International Conference on Semantic Computing (ICSC).

[56] Ying W., Xiang R., Lu Q. Improving Multi-label Emotion Classification by Integrating both General and Domain Knowledge. Proceedings of the 5th Workshop on Noisy User-Generated Text (W-NUT 2019).

[57] Kant N., Puri R., Yakovenko N., Catanzaro B. (2018). Practical Text Classification With Large Pre-Trained Language Models. arXiv.

